# Supplementary material for: Antibody-displaying extracellular vesicles for targeted cancer therapy
Source: Nat Biomed Eng. 2024 May 20;8(11):1453–68. doi: 10.1038/s41551-024-01214-6 (PMC11584392; doi:10.1038/s41551-024-01214-6)
Supplement: Supplementary file 1 — Supplementary Figures. [file 41551_2024_1214_MOESM1_ESM.pdf]

nature biomedical engineering

Article https://doi.org/10.1038/s41551-024-01214-6

Antibody-displaying extracellular vesicles for targeted cancer therapy

In the format provided by the authors and unedited

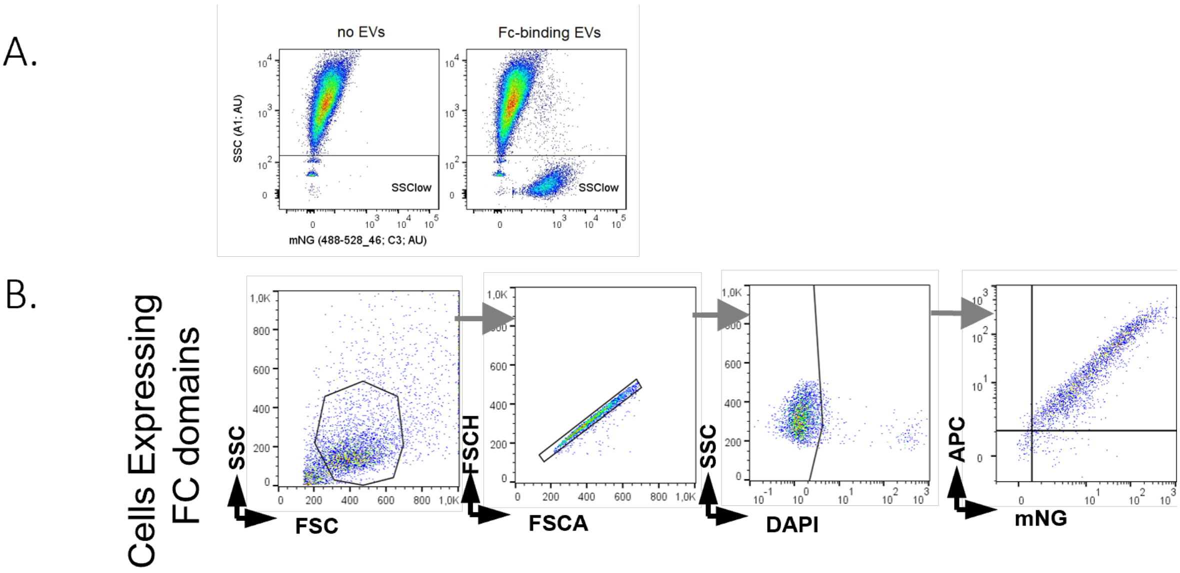

Supplementary Fig. 1 I Gating strategy of Fc-EVs and Fc-expressing cells. A. Single EV imaging flow cytometry, where data were pre-gated on SSC (low) based on mNG-tagged biological reference material described extensively before[1]. Example for pre-gating SSClow events for non-EV buffer control and EV sample using the Amnis Cellstream instrument before further gating on fluorescently labelled subsets of EVs, for further details see [1,2] B. For cells expressing Fc-binding domains, data were first gated on FSCA/SSC-A using scatter-based gating to identify cells of interest based on size and granularity, followed by scatter-based gating on FSC-A/FSC-H for identifying single cells. Next, dead cells were excluded based on DAPI staining, and viable cells of interest were further analysed for their expression of FC binding domains with EV sorting domains using REA isotype control APC-conjugated antibodies.

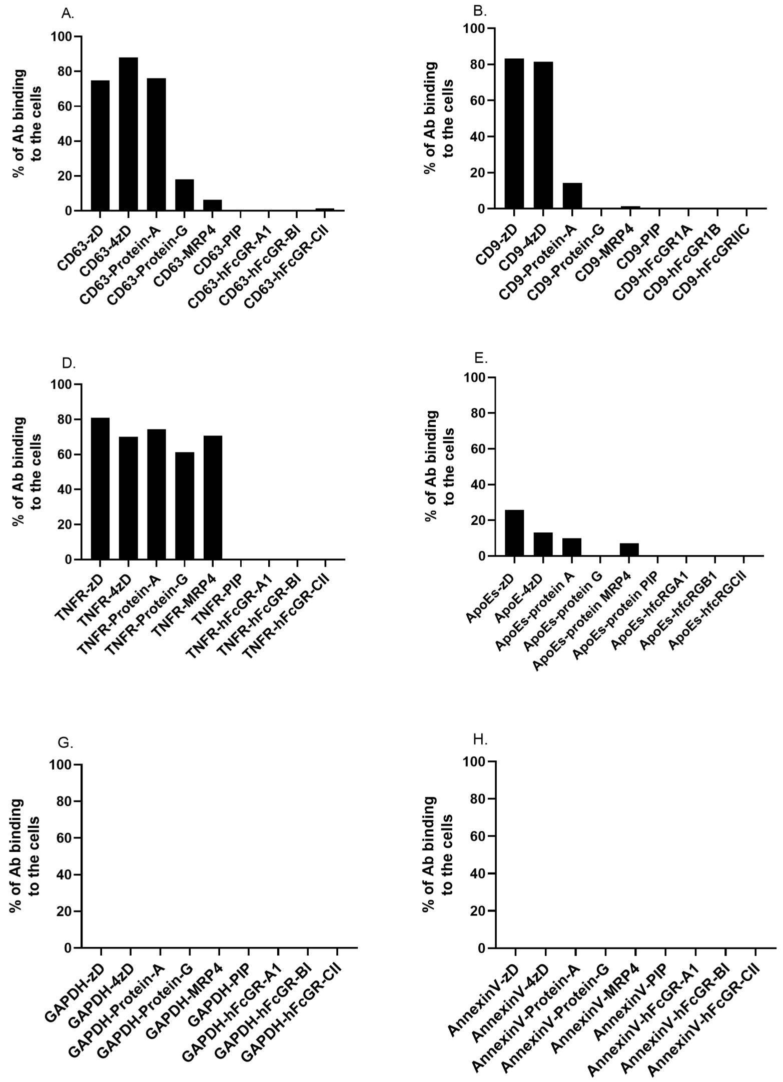

Supplementary Fig. 2 I Screen of EV sorting and Fc-binding domains. HEK293 cells transiently transfected with the 9 different EV sorting domains with C-terminal fusion of mNG and fusion with the 9 different Fc-binding domain (Fig. 1A-C). A-I show the percent binding of IgG APC to the cells as determined by flow cytometry. A) CD63-mNG + 9 different Fc binding domains. B) CD9-mNG + 9 different Fc binding domains. C) CD81-mNG + different 9 Fc binding domains. D) TNFR-mNG + 9 different Fc binding domains. E) APOE-S-mNG + 9 different Fc binding domains. F) APOE-L-mNG + 9 different Fc binding domains. G) GAPDH-mNG + 9 different Fc binding domains. H) Annexin V-mNG + 9 different Fc binding domains. I) ARRDC1-mNG + 9 different Fc binding domains.

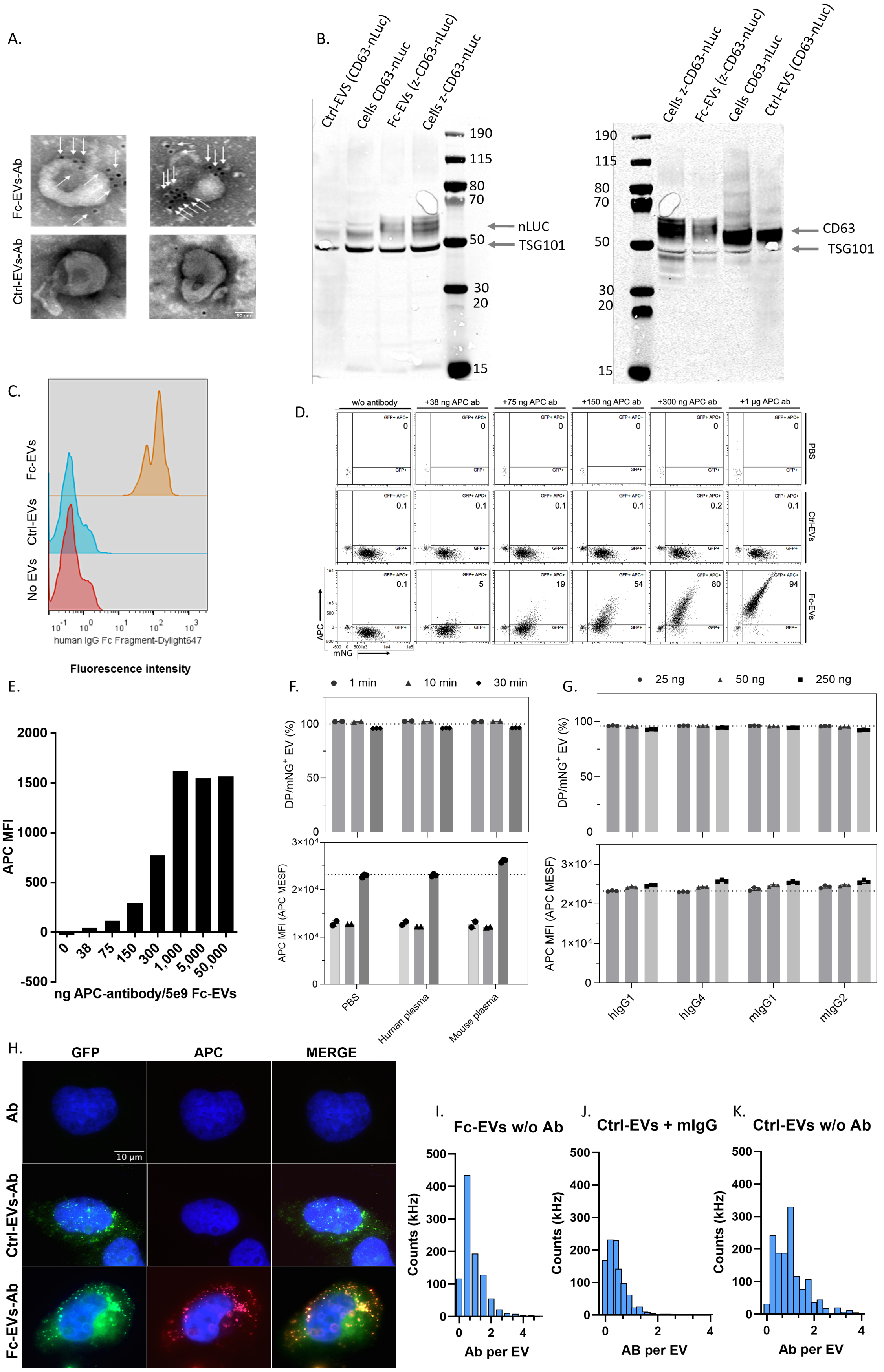

Supplementary Fig. 3 l Characterisation of Fc-EVs. A) Transmission Electron Microscopy (TEM) of nanogold particle-labelled Ab binding to Fc-EVs, but not to the control EVs (lacking the Fc-binding domain). Scalebar indicating 50 nm. B) The western blot for TSG101, CD63, and nLuc of control and Fc-EVs and their respective cell source. C) Flow cytometry showing the mean fluorescent intensity of Daylight 647 human IgG

Fc fragments incubated with Fc-EVs, control EVs, or without EVs. D) Single vesicle flow cytometry plots of APC (y-axis) and GFP (x-axis) of increasing doses of APC^{+} Ab incubated with either control- or Fc-EVs. E) Quantification of obtained APC mean fluorescence intensities following staining of Fc EVs with increasing doses of human isotype control IgG antibodies. F-G) Upper panel showing stability of binding of mNG^{+} Fc-EVs with 25 ng APC^{+} hIgG (expressed as % double positive (APC and mNG) over mNG, analysed by IFC) after subsequent exposure to F) human and mouse plasma for 1, 10, or 30 minutes, or G) PE-labelled IgG subtypes at increasing concentrations for 30 minutes. Lower panel showing MFI of APC. H) Cellular uptake of APC-labelled IgG (Ab) alone, or after incubation with mNG^{+} control- or Fc-EVs into HeLa cells. Scalebar indicating 10 μm. I-K) Quantification of antibodies per EVs was performed using the single particle profiler (SPP). I) Fc-EVs without Abs. J) Ctrl-EVs with Abs. K) Ctrl-EVs without Abs.

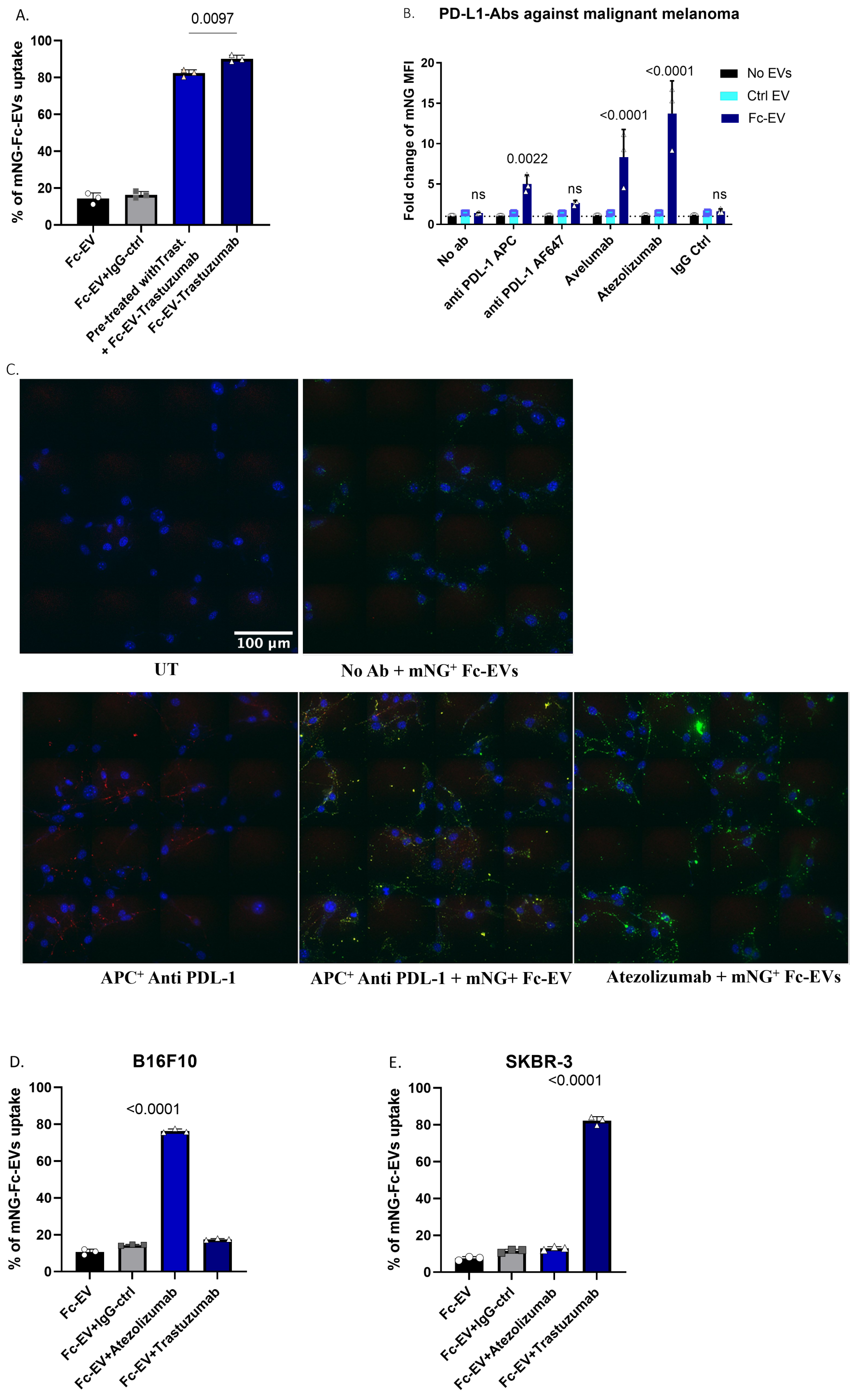

Supplementary Fig. 4 I Fc-EV targeting using PD-L1-Ab. A. Flow cytometry measurement of uptake (by %) of mNG+ Fc-EVs displaying no Ab, IgG-ctrl, or Trastuzumab with or without pre-treatment with naked Trastuzumab. B) Flow cytometry measurement of uptake (by MFI) of mNG+ EVs (Fc-EVs vs. ctrl-EVs vs. no EVs) in malignant melanoma (B16F10) cells with or without different PD-L1-Ab or control Ab (IgG Ctrl), showing the highest EV uptake of Fc-EVs with the PD-L1-Ab Atezolizumab. C) Fluorescence microscopy of B16F10 cells stained with DAPI (blue), untreated (UT), or treated with APC+ (red) PD-L1-Ab alone, or mNG+ (green) Fc-EVs alone or with APC+ PD-L1-Ab or with the PD-L1-Ab Atezolizumab, showing increased uptake of the Fc-EVs when decorated with PD-L1-Ab. Scalebar indicating 100 μm. D-E. Flow cytometry measurement of uptake (by %) of mNG+ Fc-EVs displaying no Ab, IgG-ctrl, Atezolizumab (anti-PD-L1), or Trastuzumab (anti-HER2) in D) malignant melanoma (B16F10) and E) HER2+ breast cancer cells (SKBR-3). A, B, D, E) are shown as mean ± s.d. n = 3 biological replicates. Statistical significance was calculated using one way analysis of variance (ANOVA) with Tukey's (A) or Dunnett's (D-E) post-test two-way ANOVA with Dunnett's post-test (B) as compared with each value; P values are indicated above the plots throughout.

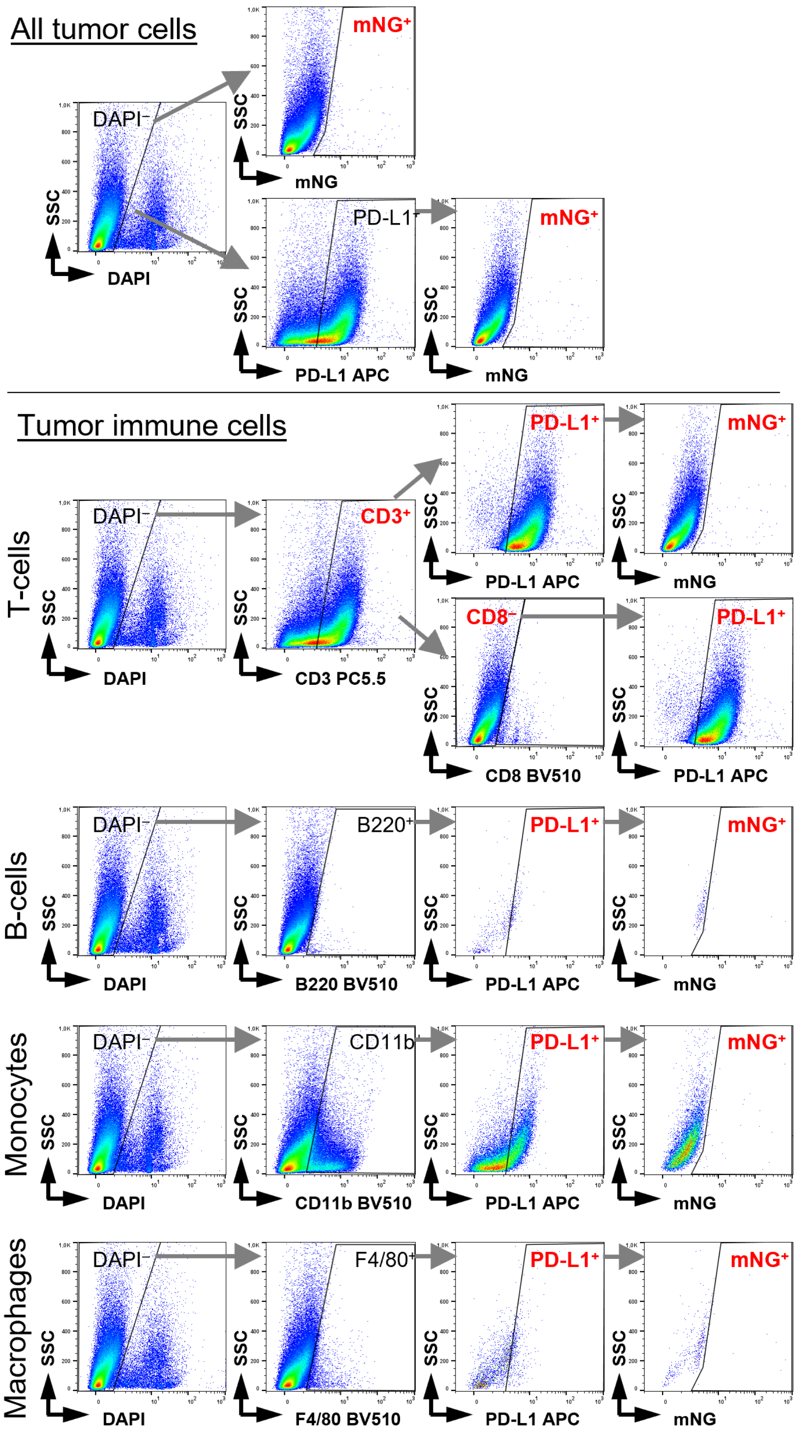

Supplementary Fig. 5 I Gating strategy of tumour cells and tumour immune cells. For all cells, data were first gated on FSC-A/FSC-H for identifying single cells, followed by scatter-based gating on FSCA/SSC-A to identify cells of interest based on size and granularity. Next, dead cells were excluded based on DAPI staining, and viable cells of interest were further analysed for their expression of CD3, CD8, B220, CD11b, F4/80+, and/or PD-L1, respectively. Here displaying the gating strategy for flow cytometry analysis of EV uptake in vivo into tumour cells and tumour immune cells, exemplified on an animal from the Fc-EV+PDL1-Ab group. Grey arrows lead the gating strategy, red labelled gates contain respective data presented in Fig. 5H-J and S6.

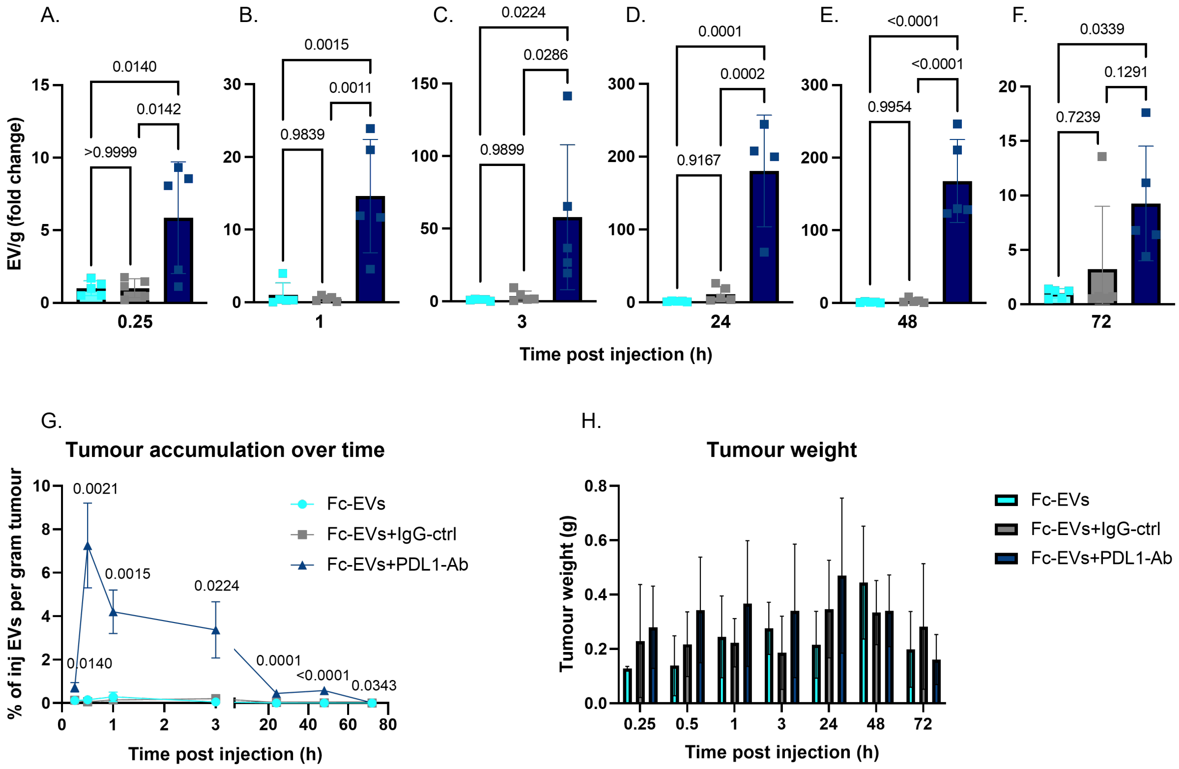

Supplementary Fig. 6 I Accumulation over time of Fc-EVs in B16F10 tumours following IV injection of PD-L1-targeted Fc-EV. Intravenous (IV) injection of Fc-EVs with PD-L1-Ab (Fc-EV+PD-L1-Ab), control-Ab (Fc-EV+IgG-ctrl), or without Ab (Fc-EV) into malignant melanoma (B16F10) tumour bearing mice. A-F) Fold change of detected EVs in tumour at indicated timepoints (below each figure) post injection.G. Percentage tumour accumulation of injected Fc-EVs (based on luminescence) per gram tumour tissue of injected dose Fc-EVs. H) Tumour weight in female C57BL/6 mice bearing B16F10. Statistical significance was calculated using one-way analysis of variance (ANOVA) with Tukey's post-test as compared with each value; If not indicated, P-values were not significant.

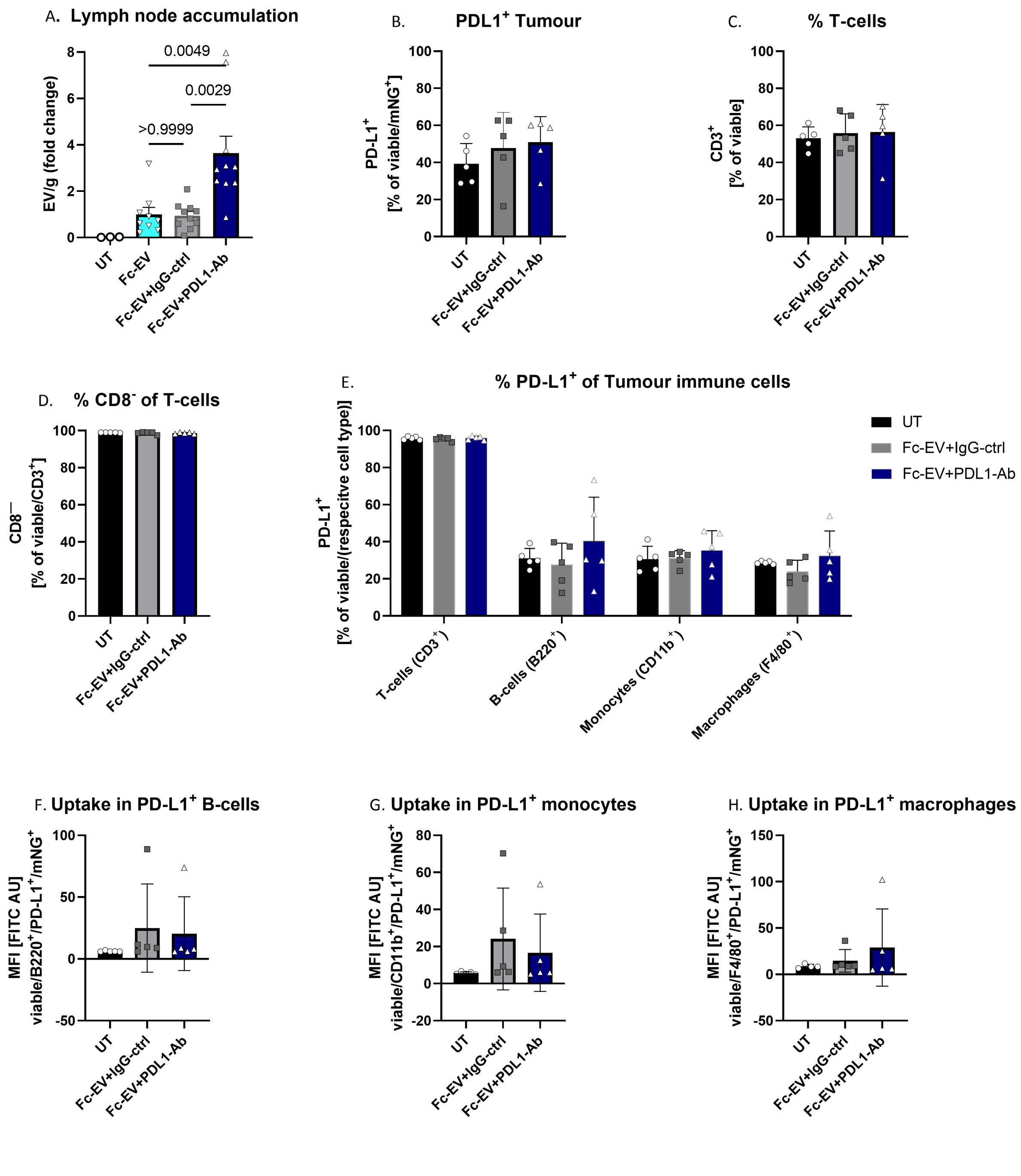

Supplementary Fig. 7 I Accumulation at 30 min post injection of Fc-EVs in B16F10 metastatic lymph nodes and tumour microenvironment following IV injection of PD-L1-targeted Fc-EV. A) Regional (metastatic) lymphnode accumulation. B-E) Flow cytometry analysis of single cell suspensions of tumour tissue following IV injection of mNG+ Fc-EV with PD-L1- Ab or control-Ab. B) Percentage PD-L1+ tumour cells that that have taken up mNG+ Fc-EVs. C) Percentage T-cells (CD3+) in tumour tissue. D) Percentage CD8- cells of the CD3+ (T-cell) cell population. E) Percentage PD-L1-positivity of the respective immune cell type. F-H) PD-L1+ immune cells that that have taken up mNG+ Fc-EVs of F) B-cells (B220+), G) monocytes (CD11b+), H) macrophages (F4/80+). Statistical significance was calculated using one-way analysis of variance (ANOVA) with Tukey's post-test as compared with each value; If not indicated, P-values were not significant.

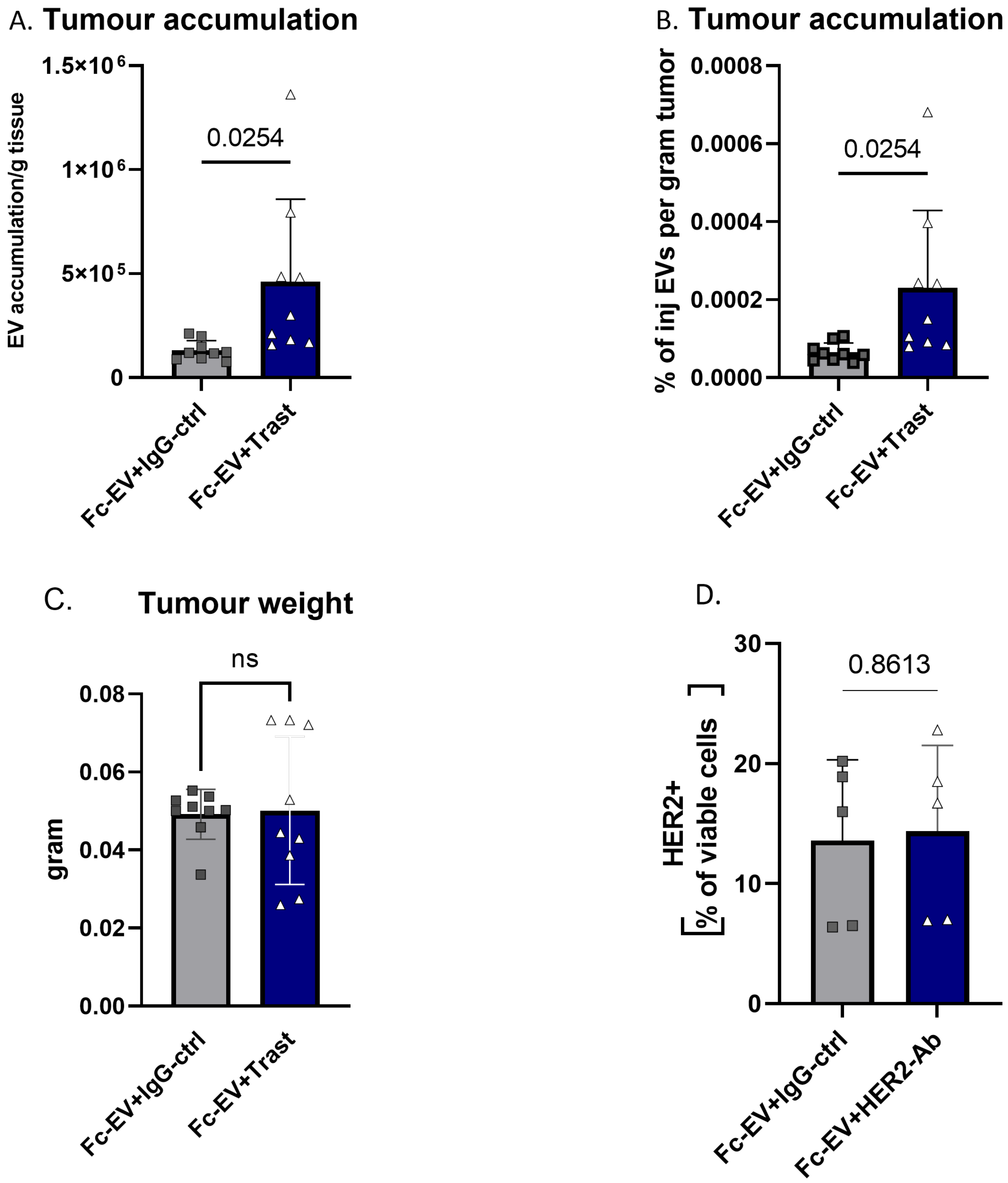

Supplementary Fig. 8 I Accumulation of Fc-EVs in SKBR-3 tumours following IV injection of HER2-targeted mNG+ Fc-EV. Intravenous (IV) injection of Fc-EVs with HER2-Ab (Trastuzumab, Fc-EV+HER2-Ab) or control-Ab (Fc-EV+IgG-ctrl) into Swiss nude mice bearing HER2+ breast cancer (SKBR-3). A) Tumour accumulation of Fc-EV+HER2- Ab (based on luminescence) per gram tumour tissue compared to Fc-EV+IgG-ctrl. B) Percentage of tumour accumulation of Fc-EV+PD-L1- Ab (based on luminescence) per gram tumour tissue compared to Fc-EV+IgG-ctrl. C) Tumour weight (in gram) of Swiss Nude mice bearing SKBR-3 after 60 days. D) Percentage of HER2 positive cells from single cell suspension of tumour tissue using flow cytometry. All data is shown as mean±s.d. n=5-10 biological replicates. Statistical significance was calculated using two-tailed unpaired t-test analysis as compared with each value; P values are indicated above the plots throughout.

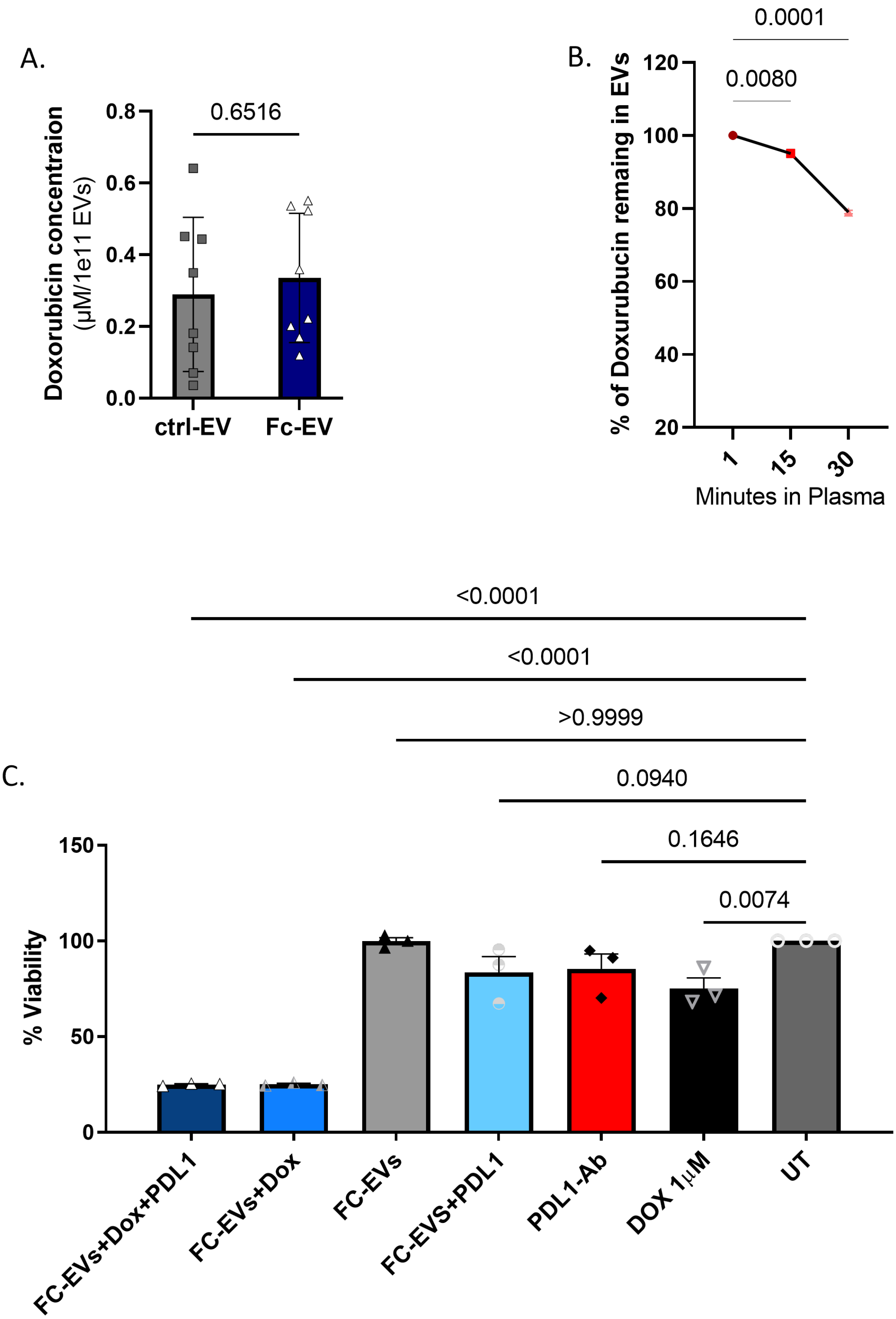

Supplementary Fig. 9 | Electroporation of Fc-EVs. A) concentration of doxorubicin (Dox) encapsulated in EVs following electroporation based on fluorescence in correlation to the standard curve of Dox in the same electroporation solution. B) Stability of encapsulated Dox loaded Fc-EVs in mouse plasma at three different time points (1, 15, and 30 min). C) Viability of B16F10 cells after 48 hours of incubation with Dox loaded Fc-EVs with (Fc-EVs+Dox+PDL1) or without PD-L1-Ab (Fc-EV+Dox) compared to unloaded Fc-EVs with (Fc-EVs+PD-L1) or without PD-L1-Ab (Fc-EV) and naked Dox. The data is shown as mean±s.d. n=3 or 8 biological replicates. Statistical significance was calculated using two-tailed unpaired t-test analysis (A) or one-way analysis of variance (ANOVA) with Dunnett's post-test (B-D) as compared with each value. P values are shown above the plots throughout.

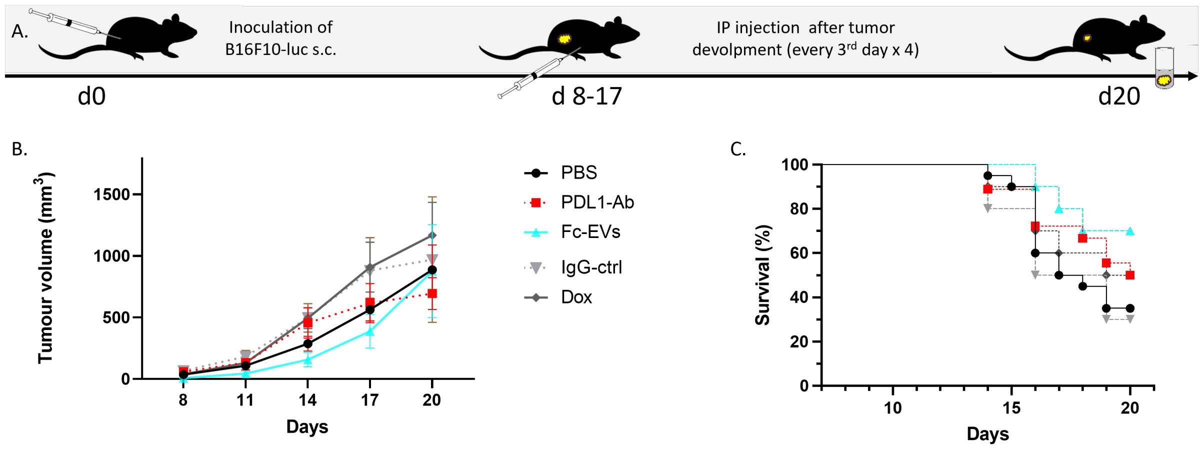

Supplementary Fig. 10 | Tumour treatment with doxorubicin loaded Fc-EVs displaying PD-L1-Ab. A) Illustration of the experimental set up. Mice were inoculated with malignant melanoma (B16F10) cells at day 0. After tumour formation, mice were treated every third day for four cycles until day 20. B-C). Tumour volume (B) and survival (C) over time for mice treated with a single entity of mock treatment (PBS), PD-L1-Ab, Fc-EVs, control-Ab (IgG-ctrl), or doxorubicin (Dox). All data is shown as mean ± s.d. n = 10-20 mice.

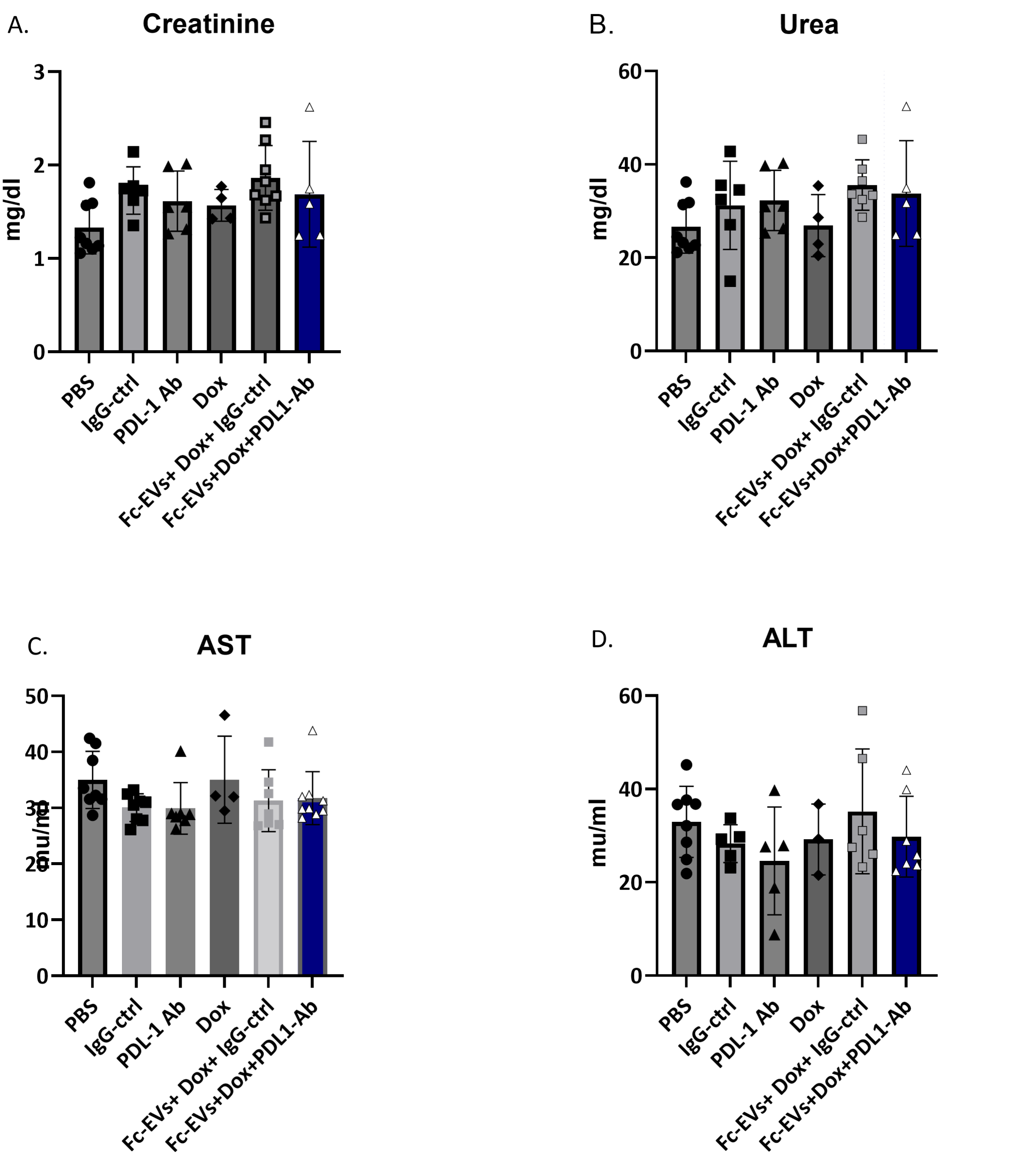

Supplementary Fig. 11 | Kidney and liver function test. A) Creatinine level in serum. B) Urea level in serum. C) Aspartate Transferase (AST) level in serum. D) Alanine transaminase (ALT) level in serum. Mice bearing tumours treated with mock treatment (PBS), control-Ab (IgG-ctrl), Atezolizumab (PD-L1-Ab), doxorubicin (Dox), control-Ab (Fc-EVs + Dox + IgG-ctrl), Dox loaded Fc-EVs with PD-L1-Ab Ab (Fc-EVs + Dox + PD-L1-Ab). The data is shown as mean ± s.d. n = 8 or 4 biological replicates. Statistical significance was calculated using one-way analysis of variance (ANOVA) with Dunnett’s post-test as compared with each value; P values are not significant.

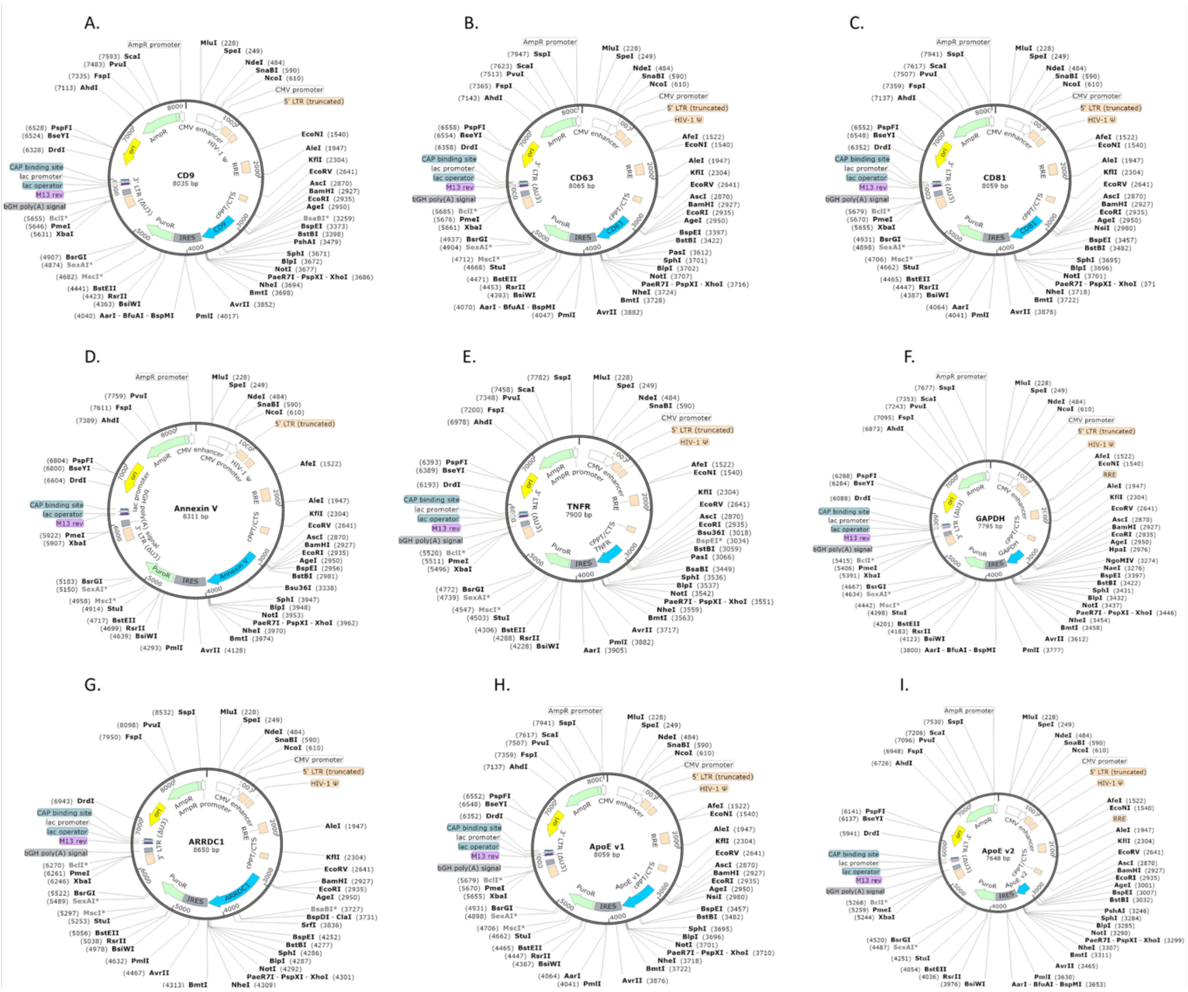

Supplementary Fig. 12 | Engineering strategy for 9 different EV sorting domains. A. CD9 cloned into p2CL9IPwo5 backbone. B. CD63 cloned into p2CL9IPwo5 backbone. C. CD81 cloned into p2CL9IPwo5 backbone. D. Annexin V cloned into p2CL9IPwo5 backbone. E. TNFR cloned into p2CL9IPwo5 backbone. F. GAPDH cloned into p2CL9IPwo5 backbone. *G.* ARRDC1 cloned into p2CL9IPwo5 backbone*.* H. ApoE v1 cloned into p2CL9IPwo5 backbone*.* I*.* ApoE v2 cloned into p2CL9IPwo5 backbone. All the fragments cloned into p2CL9IPwo5 backbone using EcoR1 and Not1 restriction enzyme. Created with BioRender.com.

A.

Human FCGR1A

```
aaatatataattcaaggtaccGAATTCgccaccATGtccggaCAGGTAGACACCACCAAAGCTGTCATCACTCTC
CAACCACCCTGGGTCAGTGTGTTTCAGGAGGAGACTGTTACACTCCATTGTGAAGTGCTGCACCT
GCCAGGTTCAAGCTCTACGCAGTGGTTTCTTAACGGGACGGCAACCCAAACCTCTACGCCTAGC
TACCGAATTACTTCAGCGTCAGTAAATGATTCCGGCGAATACAGGTGTCAGCGGGGCCTCTCTGG
ACGGTCCGACCCAATACAACTTGAGATTCACAGAGGTTGGCTGCTCTTGCAAGTATCTAGTCGCG
TATTCACAGAGGGAGAACCACTTGCCTTGCGATGTCACGCTTGGAAGGATAAGCTGGTATATAAC
GTGCTGTATTACAGAAATGGGAAAGCTTTCAAATTTTTTCACTGGAACTCCAATCTGACAATTCTTA
AGACCAACATTAGCCACAATGGTACATATCACTGTAGTGGAATGGGAAAACACAGGTACACATCA
GCCGGCATAAGTGTTACTGTAAAGGAACTGTTTCCCGCGCCTGTATTGAATGCAAGTGTCACTTC
ACCACTTCTCGAAGGGAATCTTGTGACCCTGTCTTGCGAGACGAAACTGCTCCTGCAACGACCC
GGTCTTCAACTCTACTTCTCCTTCTATATGGGTAGCAAGACTCTGCGCGGCCGAAACACATCTAG
CGAATATCAAATTCTGACGGCTAGACGCGAGGATTCTGGGCTTTATTGGTGTGAGGCAGCGACA
GAAGATGGAAATGTCTTGAAGAGGTCCCCGGAGTTGGAACTCCAGGTACTTGGACTCCAGTTGC
CAACACCTGTTTGGTTCCACTtcgaaTGAGCGGCCGCctcgagtgggaaattttaacca
```

B.

Human FcGRIB

```
aaatatataattcaaggtaccGAATTCgccaccATGtccggaCAAGTAGACACTACAAAAGCAGTCATCACATTGC
AGCCTCCCTGGGTTAGTGTCTTTCAGGAGGAAACTGTCACACTCCACTGTGAAGTGCTCCACCTT
CCGGGTAGTTCATCCACTCAATGGTTTCTCAATGGAACTGCTACTCAAACATCAACGCCTAGCTAC
AGAATTACTTCCGCGTCAGTGAACGACAGCGGAGAGTACAGGTGTCAGCGCGGGCTGTCCGGGA
GGTCAGATCCGATACAACTTGAAATCCATCGAGGTTGGCTTCTCCTGCAAGTAAGTAGCAGGGTCT
TTATGGAGGGTGAGCCCCTCGCCCTGAGGTGTCATGCGTGGAAAGACAAACTTGTGTATAATGTC
CTCTACTATAGGAACGGCAAAGCGTTCAAGTTCTTCCATTGGAACAGTAACCTTACAATCTTGAAAA
CCAATATCTCACACAATGGTACGTACCATTGTTCCGGGATGGGCAAACATAGGTACACCAGCGCAG
GAATATCACAATACACGGTTAAGGGCCTCCAATTGCCTACCCCCGTTTGGTTCCACTtcgaaTGAGC
GGCCGCctcgagtgggaaattttaacca
```

C.

Human FcgrIIC

```
aaatatataattcaaggtaccGAATTCgccaccATGtccggaACTCCCGCAGCACCGCCGAAGGCGGTGCTGAA
GCTGGAACCCCAGTGGATCAATGTGCTGCAAGAAGACTCCGTAACTCTGACCTGCCGGGGAACT
CATAGCCCCGAGAGCGATTCAATTCAATGGTTTCATAACGGCAACCTTATCCCGACTCACACACA
ACCAAGTTATAGGTTTAAGGCAAATAATAATGATTCAGGAGAATACACTTGTCAAACAGGACAAAC
AAGCCTTAGCGATCCCGTCCATCTCACGGTACTGTCCGAGTGGTTGGTTCTTCAGACGCCACACC
TTgagTTCCAAGAAGGGGAGACGATAGTGCTTAGATGCCATAGTTGGAAAGACAAGCCCCTGGTT
AAAGTTACCTTCTTTCAGAACGGTAAATCTAAAAAGTTCAGCCGCAGCGACCCAAACTTCAGCATA
CCTCAGGCGAACCATTCCCACTCCGGGGATTATCACTGTACGGGGAATATAGGATATACCCTGTA
CTCCTCCAAACCGGTTACTATAACTGTTCAAGCACCCTCCAGTTCCCCAATGGGGTtcgaaTGAGC
GGCCGCctcgagtgggaaattttaacca
```

D.

Streptococcus sp. Protein G

```
aaatatataattcaaggtaccGAATTCgccaccATGtccggaGTGGATAGTCCGATTGAAGACACTCCCATCATT
AGAAATGGAGGAGAGCTTACCAATCTTCTGGGCAATTCCGAGACAACCCTCGCCCTTAGAAACGA
AGAAAGCGCAACGGCTGATCTTACTGCCGCTGCTGTCGCTGACACGGTAGCTGCTGCAGCAGCC
GAAAATGCCGGGGCcGCCGCTTGGGAAGCCGCGGCGGCTGCTGACGCACTCGCAAAGGCAAAG
GCGGATGCTCTTAAAGAGTTCAACAAATATGGGGTATCAGATTACTACAAGAATTTGATAAATAAT
GCCAAGACAGTGGAAGGGGTCAAAGATCTGCAGGCGCAAGTGGTAGAAAGCGCCAAAAAGGCG
CGCATTTCCGAAGCAACGGACGGACTTAGTGATTTCCTCAAAAGTCAAACTCCTGCAGAGGATAC
GGTCAAAAGTATAGAGTTGGCTGAAGCAAAAGTTTTGGCAAATAGAGAACTGGATAAATATGGTG
TCTCCGATTATCACAAAAACTTGATAAACAACGCCAAAACCGTTGAAGGTGTAAAAGACCTCCAG
GCCCAAGTAGTGGAGTCTGCGAAGAAAGCTCGAATAAGCGAGGCTACAGATGGTCTGAGCGATT
TTTTGAAGTCACAAACACCGGCTGAAGACACTGTTAAATCCATAGAACTCGCAGAAGCAAAAGTC
CTTGCTAACAGAGAGCTCGATAAGTATGGTGTCTCAGACTACTACAAGAACCTGATCAATAATGC
GAAGACAGTGGAGGGGGTTAAGGCCCTCATTGACGAGATACTGGCAGCACTGCCTAAAACGGAT
ACTTACAAGTTGATTCTCAATGGCAAAACACTTAAGGGTGAAACTACGACCGAAGCCGTAGATGC
TGCGACTGCCGAAAAGGTATTTAAACAATACGCCAACGATAACGGCGTCGACGGCGAGTGGACG
TACGACGATGCAACCAAAACTTTTACTGTCACGGAGAAGCCTGAAGTCATAGATGCCTCCGAATT
GACTCCCGCAGTCACTACGTATAAACTTGTTATTAATGGGAAGACACTTAAGGGTGAGACCACCA
CTGAGGCCGTCGATGCTGCGACAGCTGAGAAAGTCTTCAAGCAATACGCAAACGATAACGGTGT
TGACGGGGAATGGACCTACGACGATGCCACCAAGACATTTACGGTCACCGAAAAGCCGGAGGTT
ATTGATGCCTCCGAACTTACTCCTGCTGTGACGACATACAAGTTGGTTATAAACGGTAAGACTCTT
AAAGGTGAGACTACGACTAAAGCAGTTGACGCTGAAACTGCCGAAAAGGCCTTCAAGCAGTATG
CGAATGACAATGGGGTAGACGGTGTATGGACCTACGACGATGCTACCAAGACCTTCACGGTAAC
TGAAATGGTGACTGAGGTGCCAGGCGATGCGCCTACCGAACCTGAGAAACCCGAAGCCTCTATA
CCCCTGGTtCCCCTGACACCTGCAACACCCATTGCGAAGGATGACGCAAAAAAGGACGACACTAA
AAAGGAAGACGCCAAAAAGCCAGAAGCTAAAAAGGAAGATGCAAAGAAAGCTGAAACCTTGCCTA
CTACGTtcgaaTGAGCGGCCGCctcgagtgggaaattttaacca
```

E.

Streptococcus sp. MRP4

```
aaatatataattcaaggtaccGAATTCgccaccATGtccggaGAATCCAGAAGATACCAGGCTCCCCCGCGCGT
GTTGCTTCAGGGAAAAGAGGCCAATAAGGTCTTTGAAGAAAGGAAGGCACTTGAAAAGCAGGCG
AGGGATCTGGGAGATACGATCAATCATATGAGCCAGACTATAAGTGAACAGAGTAGGAAGATTGC
CGCCCTGAAATCTGAAGCGGAACTCAAGAATCAGCAGGCCCTTGAGGCGCTGAACAACAAGAAT
AAGCAAATCAGTGATCTCACGAATGAAAATGCCCAACTGAAGGAAGCGATCGAAGGCTACGTGCA
AACAATCCAGAATGCATCTAGGGAAATAGCCGCCAAGCAACAAGAGTTGGCTGCTGCAAAATCTC
AACTTGAAGCCAAGAACGCGGAGATTGAAGCCCTTAAACAGCAGGATGCGTCAAAGACCGAGGA
AATCGCTAAACTCCAGTCAGAGGCTGCGACTCTGGAGAATCTCTTGGGATCAGCCAAGCGGGAG
CTGACCGAACTCCAGGCCAAGCTCGATACCGCAACAGCGGAAAAGGCCAAACTGGAGTCACAGG
TGACTACTTTGGAGAATCTTTTGGGCTCCGCGAAACGCGAGCTCACAGATTTGCAGGCTAAACTG
GACGCTGCAAATGCAGAGAAGGAAAAACTGCAATCCCAAGCTGCGACGCTGGAGAAGCAATTGG
AAGCGACGAAGAAAGAACTTGCAGATCTTCAGGCCAAGCTCGCCGCGACTAACCAAGAGAAGGA
GAAATTGGAGGCCGAAGCTAAGGCACTGAAAGAGCAACTCGCAAAACAAGCAGAGGAGCTGGCT
AAGTTGAAGGCGGACAAAGCGTCTGGCGCACAAAAGCCAGACACAAAACCAGGCAACAAAGAAG
TACCTACCCGGCCGTCACAGACGCGGACCAATACGAACAAAGCCCCTATGGCACAAACCAAGCG
CCAGCTCCCCAGTACATtcgaaTGAGCGGCCGCctcgagtgggaaattttaacca
```

F.

Staphylococcus Aureus Protein A

```
aatatataattcaaggtaccGAATTCgccaccATGtccggaGCCCAGCATGATGAGGCGCAGCAGAACGCCTTTT
ACCAGGTCTTGAATATGCCAAACTTGAATGCAGATCAGCGGAACGGATTCATACAAAGCCTTAAAG
ATGATCCTTCTCAATCTGCAAATGTTCTCGGTGAAGCGCAGAAGCTTAACGATTCCCAGGCCCCTA
AGGCAGATGCCCAACAGAATAACTTCAACAAAGACCAACAGTCTGCTTTTTACGAAATCCTGAACA
TGCCTAATCTGAACGAAGCACAACGCAATGGGTTCATTCAAAGTTTGAAGGACGATCCTAGCCAAA
GCACTAACGTACTCGGTGAGGCCAAAAAGCTGAACGAATCCCAAGCGCCCAAGGCAGATAACAAT
TTTAATAAAGAACAGCAAAATGCTTTCTATGAGATTCTGAATATGCCAAACCTTAATGAAGAGCAACG
AAACGGTTTTATCCAAAGCCTCAAAGATGACCCTAGCCAAAGTGCTAACTTGCTCTCCGAAGCTAA
GAAGCTGAATGAGTCTCAAGCACCGAAAGCAGATAATAAATTCAATAAAGAGCAGCAAAACGCATT
CTATGAAATCTTGCATTTGCCCAACCTTAATGAAGAACAGCGCAACGGGTTTATTCAGTCTCTCAAA
GACGATCCCTCCCAGAGTGCCAACTTGCTGGCGGAGGCCAAAAAACTTAATGACGCGCAGGCGC
CCAAGGCGGATAACAAGTTTAACAAGGAGCAGCAAAACGCGTTTTATGAAATCCTGCACCTCCCCA
ACCTTACCGAGGAACAACGAAATGGCTTTATACAAAGTCTCAAGGATGACCCATCTGTATCCAAGG
AAATTCTTGCGGAGGCCAAGAAACTTAACGACGCGCAAGCCCCTAAATtcgaaTGAGCGGCCGCctc
gagtgggaaattttaacca
```

G.

4z domain (from Protein A)

```
aaatatataattcaaggtaccGAATTCgccaccATGtccggaGTAGACAACAAATTTAACAAGGAACAACAGAATG
CATTCTATGAGATTCTTCACCTGCCGAACCTGAATGAAGAGCAACGCAACGCCTTCATTCAATCAT
TGAAGGACGACCCGTCTCAAAGCGCAAACCTCCTTGCGGAAGCGAAGAAACTCAATGATGCTCA
GGCGCCAAAAGGAAGTGGATCCGGGTCCGGGAGCGGTTCTGTCGATAACAAATTTAACAAAGAG
CAACAGAATGCCTTTTATGAAATTCTTCATCTGCCTAATCTCAATGAAGAGCAACGGAATGCGTTT
ATACAAAGCCTCAAAGATGATCCAAGTCAGTCAGCGAACCTCCTTGCCGAGGCTAAAAAGCTCAA
TGATGCACAAGCGCCTAAGGGAAGCGGTTCAGGAAGCGGAAGCGGCTCCGTCGACAATAAGTTC
AATAAAGAACAGCAGAATGCTTTCTACGAGATTCTCCACCTTCCTAATTTGAACGAGGAGCAAAG
GAACGCTTTCATCCAAAGTCTTAAAGATGATCCGTCTCAAAGTGCCAATCTGTTGGCCGAAGCGA
AAAAATTGAACGACGCGCAAGCGCCGAAGGGAAGCGGGTCAGGATCCGGCAGTGGATCAGTGG
ATAATAAATTTAATAAAGAACAACAGAATGCCTTCTACGAGATATTGCACTTGCCCAACCTTAATGA
AGAACAGAGGAACGCATTCATACAATCACTTAAAGACGATCCCTCCCAGTCCGCCAATTTGCTGG
CTGAAGCTAAGAAATTGAACGACGCCCAAGCTCCAAAGTtcgaaTGAGCGGCCGCctcgagtgggaaatttt
aacca
```

H.

Z domain (from Protein A)

```
aaatatataattcaaggtaccGAATTCgccaccATGtccggaGTAGACAACAAATTTAACAAGGAACAACAGAATG
CATTCTATGAGATTCTTCACCTGCCGAACCTGAATGAAGAGCAACGCAACGCCTTCATTCAATCAT
TGAAGGACGACCCGTCTCAAAGCGCAAACCTCCTTGCGGAAGCGAAGAAACTCAATGATGCTCA
GGCGCCAAAATtcgaaTGAGCGGCCGCctcgagtgggaaattttaacca
```

I.

PIP

```
aaatatataattcaaggtaccGAATTCgccaccATGtccggaCAGGATAACACACGGAAGATCATCATCAAGAAT
TTTGATATACCAAAATCAGTTAGGCCCAATGACGAAGTGACGGCAGTGCTGGCAGTCCAGACTGA
ATTGAAGGAATGTATGGTGGTCAAGACGTATCTGATTTCATCCATACCGCTCCAGGGTGCCTTCA
ATTACAAATACACAGCGTGCCTGTGTGATGACAACCCGAAGACATTCTACTGGGATTTTTATACCA
ACCGCACTGTCCAAATCGCCGCCGTGGTGGACGTAATCAGAGAACTGGGGATCTGCCCCGACGA
CGCGGCTGTAATCCCAATAAAGAACAACAGGTTCTACACCATCGAAATCCTCAAAGTGGAgTtcgaa
TGAGCGGCCGCctcgagtgggaaattttaacca
```

J.

mNeon Green (mNG)

```
aaatatataattcaaggtaccGAATTCgccaccATGgcatgcGTGTCAAAGGGGGAAGAAGACAATATGGCCTCA
CTTCCTGCCACACACGAGCTTCATATTTTCGGCTCTATCAACGGAGTAGACTTCGACATGGTCGG
TCAGGGAACAGGGAACCCTAACGACGGTTACGAGGAATTGAACCTCAAAAGTACAAAAGGTGAC
CTGCAATTCTCTCCTTGGATCCTTGTGCCCCACATTGGCTATGGTTTTCATCAGTACCTCCCTTAC
CCCGACGGCATGAGCCCTTTTCAAGCAGCTATGGTTGACGGATCCGGCTATCAGGTCCACCGCA
CGATGCAATTTGAGGATGGGGCCTCCCTCACTGTTAATTATCGATACACCTATGAAGGATCACAT
ATAAAAGGAGAAGCTCAAGTCAAAGGCACCGGATTTCCCGCCGATGGTCCAGTAATGACAAACA
GCCTTACCGCAGCAGACTGGTGTAGGTCAAAAAAGACATACCCAAACGATAAGACGATAATAAGC
ACTTTCAAGTGGAGTTATACCACAGGGAACGGAAAGCGtTACCGGAGCACTGCCCGGACGACGT
ACACTTTCGCAAAACCGATGGCGGCTAACTATTTGAAAAACCAACCTATGTATGTGTTCCGAAAGA
CTGAACTCAAACATTCAAAAACTGAACTGAATTTCAAGGAATGGCAGAAGGCGTTTACAGACGTG
ATGGGAATGGATGAACTTTATAAACATCATCACCATCACCATaccggtTGAGCGGCCGCctcgagtggga
aattttaacca
```

K.

Nano-luciferase (nLuc)

```
aaatatataattcaaggtaccGTCTTCACATTGGAAGACTTCGTTGGGGATTGGAGGCAGACCGCCGGGTA
TAACTTGGATCAGGTACTCGAACAAGGCGGAGTATCTTCCCTGTTCCAAAATCTGGGGGTTAGCG
TCACGCCCATTCAACGCATTGTGCTCTCCGGTGAGAATGGACTGAAGATCGATATACACGTAATT
ATCCCCTATGAGGGGCTGTCTGGTGATCAGATGGGGCAGATAGAGAAAATCTTTAAGGTAGTTTA
CCCCGTGGATGATCACCACTTCAAAGTAATCCTCCATTATGGTACACTCGTGATTGACGGGGTCA
CACCAAACATGATCGATTACTTCGGCAGACCGTATGAGGGCATAGCAGTATTCGACGGAAAGAAG
ATAACAGTCACGGGGACATTGTGGAATGGAAACAAAATAATTGATGAACGCCTCATCAACCCGGA
CGGAAGTCTTCTTTTTCGCGTCACAATCAACGGCGTGACGGGGTGGCGGCTCTGTGAAAGGATC
TTGGCGctcgagtgggaaattttaacca
```

Supplementary Fig. 13 I DNA sequence of 9 different Fc-binding domain fragments, mNeon Green (mNG) and Nano-luciferase (nluc). A. Human gamma Fc receptor 1A (FCGR1A). B. Human gamma Fc receptor 1B (FCGR1B). C. Human gamma Fc receptor IIC (FCGRIIC). D. Protein G from *Streptococcus sp*. E. Multidrug resistance protein 4 from *Streptococcus sp*. F. Protein A from *Staphylococcus aureus. G. 4Z domains* from Protein A (*Staphylococcus aureus). H. Z domain* from Protein A (*Staphylococcus aureus).* H*. Z domain* from Protein A (*Staphylococcus aureus).* I. Human Prolactin Induced Protein. p2CL9IPwo5 backbone-EVs sorting domains were used to clone all of the Fc segments using the restriction enzymes Kpn2I and Bsp119I.. J. mNG sequence K. nluc sequence. Both mNG and nluc cloned into p2CL9IPwo5 backbone-EVs sorting domains using PaeI and NotI restriction enzyme at the C-terminus of the EVs sorting domains. AgeI and EcoRI restriction enzymes were employed to clone mNG and nluc for the N-terminus of the p2CL9IPwo5 backbone-EVs sorting domains.

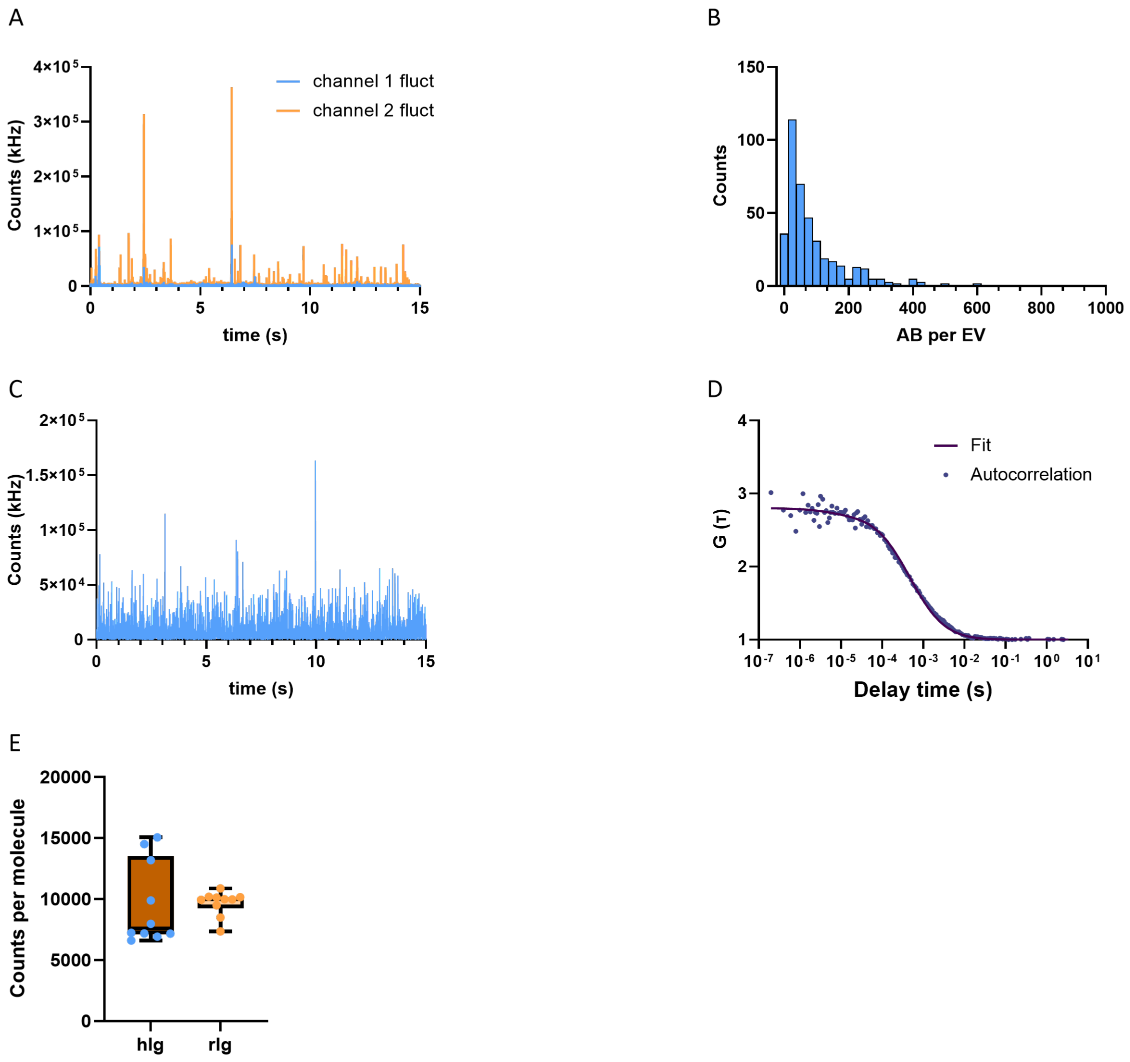

Supplementary Fig. 14 I Measurement of the number of antibodies per EV by single particle profiler[3]. a) Fluorescence fluctuation of EVs diffusing through the observation volume, single peaks represent single particles; b) Histogram of numbers of antibodies per EV; c) Fluorescence fluctuation of antibodies diffusion in water; d) Autocorrelation function of fluctuations in c; e) Fluorescence brightness of antibodies.

Supplementary references

1. Gorgens, A. et al. Optimisation of imaging flow cytometry for the analysis of single extracellular vesicles by using fluorescence-tagged vesicles as biological reference material. *J Extracell Vesicles* **8**, 1587567 (2019).
2. Gupta, D. et al. Amelioration of systemic inflammation via the display of two different decoy protein receptors on extracellular vesicles. *Nat Biomed Eng* **5**, 1084-1098 (2021).
3. Sych, T. et al. High-throughput measurement of the content and properties of nano-sized bioparticles with single-particle profiler. *Nat Biotechnol* (2023).
