# Supplementary material for: Antibody-displaying extracellular vesicles for targeted cancer therapy
Source: Nat Biomed Eng. 2024 May 20;8(11):1453–68. doi: 10.1038/s41551-024-01214-6 (PMC11584392; doi:10.1038/s41551-024-01214-6)
Supplement: Supplementary file 2 — Reporting Summary [file 41551_2024_1214_MOESM2_ESM.pdf]

nature portfolio

Corresponding author(s): Oscar Wiklander and Samir EL Andaloussi

Last updated by author(s): Apr 2, 2024

Reporting Summary

Nature Portfolio wishes to improve the reproducibility of the work that we publish. This form provides structure for consistency and transparency in reporting. For further information on Nature Portfolio policies, see our Editorial Policies and the Editorial Policy Checklist.

Statistics

For all statistical analyses, confirm that the following items are present in the figure legend, table legend, main text, or Methods section.

n/a	Confirmed	
☐	☒	The exact sample size (*n*) for each experimental group/condition, given as a discrete number and unit of measurement
☐	☒	A statement on whether measurements were taken from distinct samples or whether the same sample was measured repeatedly
☐	☒	The statistical test(s) used AND whether they are one- or two-sided<br>*Only common tests should be described solely by name; describe more complex techniques in the Methods section.*
☐	☒	A description of all covariates tested
☐	☒	A description of any assumptions or corrections, such as tests of normality and adjustment for multiple comparisons
☐	☒	A full description of the statistical parameters including central tendency (e.g. means) or other basic estimates (e.g. regression coefficient) AND variation (e.g. standard deviation) or associated estimates of uncertainty (e.g. confidence intervals)
☐	☒	For null hypothesis testing, the test statistic (e.g. *F*, *t*, *r*) with confidence intervals, effect sizes, degrees of freedom and *P* value noted<br>*Give P values as exact values whenever suitable.*
☒	☐	For Bayesian analysis, information on the choice of priors and Markov chain Monte Carlo settings
☐	☒	For hierarchical and complex designs, identification of the appropriate level for tests and full reporting of outcomes
☐	☒	Estimates of effect sizes (e.g. Cohen's *d*, Pearson's *r*), indicating how they were calculated

Our web collection on statistics for biologists contains articles on many of the points above.

Software and code

Policy information about availability of computer code

Data collection	Microsoft Excel, NanoSight NTA software, MACSQuant Analyzer 10 flow cytometer, SoftMax Pro, Amnis ImageStream X Mk II and Amnis Cellstream software. Nanoimager S Mark II microscope software.
Data analysis	Prism 8-10, Microsoft excel, FlowJo.

For manuscripts utilizing custom algorithms or software that are central to the research but not yet described in published literature, software must be made available to editors and reviewers. We strongly encourage code deposition in a community repository (e.g. GitHub). See the Nature Portfolio guidelines for submitting code & software for further information.

Data

Policy information about availability of data

All manuscripts must include a data availability statement. This statement should provide the following information, where applicable:

- Accession codes, unique identifiers, or web links for publicly available datasets
- A description of any restrictions on data availability
- For clinical datasets or third party data, please ensure that the statement adheres to our policy

The raw and analysed datasets are available from figshare at https://doi.org/10.6084/m9.figshare.25338379.

Human research participants

Policy information about studies involving human research participants and Sex and Gender in Research.

Reporting on sex and gender	The study did not involve human participants.
Population characteristics	–
Recruitment	–
Ethics oversight	–

Note that full information on the approval of the study protocol must also be provided in the manuscript.

Field-specific reporting

Please select the one below that is the best fit for your research. If you are not sure, read the appropriate sections before making your selection.

☒ Life sciences ☐ Behavioural & social sciences ☐ Ecological, evolutionary & environmental sciences

For a reference copy of the document with all sections, see nature.com/documents/nr-reporting-summary-flat.pdf

Life sciences study design

All studies must disclose on these points even when the disclosure is negative.

Sample size	Sample sizes were determined without any pre-calculations of the number needed; instead, we used educated assumptions based on previous experience of the experimental setup of the in vitro and in vivo experiments.
Data exclusions	Data from one mouse at 24h in the Fc-EVs+PDL1-Ab was excluded as an outlier (which gave unreasonably high luminescent values, confirmed using the graphpads outlier calculator at https://www.graphpad.com/quickcalcs/grubbs1).
Replication	All attempts at replication were successful.
Randomization	Divisions into the different experimental groups were conducted in each experiment, after cells had been plated or mice had been inoculated, by randomly choosing wells or mice, respectively, for each treatment group.
Blinding	No blinding of the in vitro or in vivo work was conducted. All in vivo experiments were conducted together with at least two investigators, and the measurements were divided between the researchers on different days in to minimize bias.

Reporting for specific materials, systems and methods

We require information from authors about some types of materials, experimental systems and methods used in many studies. Here, indicate whether each material, system or method listed is relevant to your study. If you are not sure if a list item applies to your research, read the appropriate section before selecting a response.

Materials & experimental systems

n/a	Involved in the study	
☐	☒	Antibodies
☐	☒	Eukaryotic cell lines
☒	☐	Palaeontology and archaeology
☐	☒	Animals and other organisms
☒	☐	Clinical data
☒	☐	Dual use research of concern

Methods

n/a	Involved in the study	
☒	☐	ChIP-seq
☐	☒	Flow cytometry
☒	☐	MRI-based neuroimaging

Antibodies

Antibodies used	rb-α-ms PD-L1 (APC, 485, Sino Biological), rt-α-ms CD3 (17A2, BioLegend), rt-α-ms CD8a (53-6.7, BioLegend), rt-α-ms CD45R/B220 (RA3.6B2, BioLegend), rt-α-ms CD11b (M1/70, BD Biosciences), rt-α-ms F4/80 (T45-2342, BD Biosciences), anti-CD63 (ab134045, Abcam), anti-Alix (MA1-83977, Thermofisher), Anti-NanoLuc (N7000, Promega), anti-Tsg101 (ab30871, Abcam), secondary antibody (Goat anti-Mouse (C00322), Goat anti-Rabbit (C90827-25), Rabbit anti-goat 10nm ab conjugated with gold nanoparticles (BBI

	Solutions), anti-CD9, Anti-CD63 and anti-CD81 detection antibodies (supplied in the MACSPlex Exosome Kit, human, Miltenyi Biotec), AlexaFluor647-conjugated human IgG Fc fragments (The Jackson Laboratory, cat 009-600-008), hIgG1-PE (Miltenyi; Cat. No. 130-113-438), hIgG4-PE (BioLegend; Cat. No. 403704), mIgG1-PE (Miltenyi; Cat. No. 130-113-200), and mIgG2-PE (Miltenyi; Cat. No. 130-092-215), Atezolizumab and Trastuzumab were kind gifts from Karolinsak University Hospital.
Validation	The validation of the antibodies had been conducted by the distributor, and they were not further validated other than as described in the paper.

Eukaryotic cell lines

Policy information about cell lines and Sex and Gender in Research

Cell line source(s)	FreeStyle 293F (HEK293FS; ThermoFisher Scientific), HEK293T (ATCC, CRL-3216), HeLa (ATCC, CCL-2), SKBR3 (ATCC, HTB-30), and B16F10 (ATCC, CRL-6475-LUC2).
Authentication	None of the cell lines were authenticated other then by morphology.
Mycoplasma contamination	All cell lines were confirmed negative by testing for mycoplasma.
Commonly misidentified lines (See ICLAC register)	No commonly misidentified cell lines were used.

Animals and other research organisms

Policy information about studies involving animals; ARRIVE guidelines recommended for reporting animal research, and Sex and Gender in Research

Laboratory animals	C57BL/6 and Swiss nude mice. All mice were kept at a dedicated laboratory animal facility with controlled temperature, light-dark cycles, with access to nesting material, food and water.
Wild animals	The study did not involve wild animals.
Reporting on sex	Only female mice were used in order to reduce the number of mice and unwanted factors, owing to the fact that male mice may fight more with each other, especially when carrying diseases such as cancer.
Field-collected samples	The study did not involve samples collected from the field.
Ethics oversight	All of the animal experiments were performed in accordance with ethical permissions approved by The Swedish Local Board for Laboratory Animals, and designed to minimize the suffering and pain of the animals.

Note that full information on the approval of the study protocol must also be provided in the manuscript.

Flow Cytometry

Plots

Confirm that:

☒ The axis labels state the marker and fluorochrome used (e.g. CD4-FITC).

☒ The axis scales are clearly visible. Include numbers along axes only for bottom left plot of group (a 'group' is an analysis of identical markers).

☒ All plots are contour plots with outliers or pseudocolor plots.

☒ A numerical value for number of cells or percentage (with statistics) is provided.

Methodology

Sample preparation	Sample preparations for either cells or EV measurements by flow cytometry are detailed in Methods.
Instrument	MACSQuant Analyzer 10 flow cytometer, Amnis ImageStream X Mk II, and Amnis Cellstream.
Software	The respective instrument's software was used to acquire the data. FlowJo was used for analysis.
Cell population abundance	Not applicable.
Gating strategy	The gating strategies are shown in the Supplementary Information. In short, for tumour-cell analysis, dead cells were excluded based on DAPI staining, and viable cells of interest were further analysed for their expression of mNG, CD3, CD8, B220, CD11b, F4/80, and/or PD-L1, respectively. For bead-based multiplex flow-cytometry analysis of EV surface markers (MACSPlex assay), single beads were identified based on FSC-H vs SSH-H and subsequent FSC-A/FSC-H gating, and fluorescent capture-bead populations were identified according to the manufacturer's recommendation and as described previously (Wiklander, Frontiers Immunol. 2018). For single-EV imaging flow cytometry, data were pre-gated on SSC(low) based on GFP-

tagged biological reference material described extensively before (Görgens,JEV 2019).

☒ Tick this box to confirm that a figure exemplifying the gating strategy is provided in the Supplementary Information.
